# Cram’s Family Atlas

**Published:** 1884-07

**Authors:** 


CRAM'S UNRIVALED FAMILY ATLAS OF THE WORLD, Hudgins & Talty, Publishers, Atlanta, Ga., 1884.

This work is without doubt the most complete and comprehensive book of the kind ever presented to the public. It contains ninety-six large maps, embracing in detail every country of modern, and many empires of ancient times; diagrams excellently arranged to show the comparative sizes of the countries of the world, population, areas, religions, races, products, wealth, debts, commerce, money circulation, accumulated wealth, strength of armies and navies, railroads and telegraphs; statistics and tables showing distances between points; statistics of life, population of cities of the United States and the world; principal nations and rulers of the world; rates of postage; tobacco production of the United States; table showing where gold and silver come from; value of land in the United States. Also, illustrations of flags, principal high buildings of the world, progress of a century, prominent species of birds, Presidents of the United States, races of man, vegetable and animal life of the five zones.

It also contains a complete index of every city, town and post-office in the United States, giving location and population.

The atlas must be seen and examined to be appreciated. It is one of few books laid before the public by canvassers worthy of notice, and should be in the hands of every scholar and on the library table of every home.

